# Self-Oscillating Curling of a Liquid Crystal Elastomer Beam under Steady Light

**DOI:** 10.3390/polym15020344

**Published:** 2023-01-09

**Authors:** Junxiu Liu, Junjie Zhao, Haiyang Wu, Yuntong Dai, Kai Li

**Affiliations:** 1 Anhui Province Key Laboratory of Building Structure and Underground Engineering, Anhui Jianzhu University, Hefei 230601, China; 2 College of Civil Engineering, Anhui Jianzhu University, Hefei 230601, China

**Keywords:** self-oscillation, liquid crystal elastomer, curling, optically-responsive, dynamic boundary problem

## Abstract

Self-oscillation absorbs energy from a steady environment to maintain its own continuous motion, eliminating the need to carry a power supply and controller, which will make the system more lightweight and promising for applications in energy harvesting, soft robotics, and microdevices. In this paper, we present a self-oscillating curling liquid crystal elastomer (LCE) beam-mass system, which is placed on a table and can self-oscillate under steady light. Unlike other self-sustaining systems, the contact surface of the LCE beam with the tabletop exhibits a continuous change in size during self-sustaining curling, resulting in a dynamic boundary problem. Based on the dynamic LCE model, we establish a nonlinear dynamic model of the self-oscillating curling LCE beam considering the dynamic boundary conditions, and numerically calculate its dynamic behavior using the Runge-Kutta method. The existence of two motion patterns in the LCE beam-mass system under steady light are proven by numerical calculation, namely self-curling pattern and stationary pattern. When the energy input to the system exceeds the energy dissipated by air damping, the LCE beam undergoes self-oscillating curling. Furthermore, we investigate the effects of different dimensionless parameters on the critical conditions, the amplitude and the period of the self-curling of LCE beam. Results demonstrate that the light source height, curvature coefficient, light intensity, elastic modulus, damping factor, and gravitational acceleration can modulate the self-curling amplitude and period. The self-curling LCE beam system proposed in this study can be applied to autonomous robots, energy harvesters, and micro-instruments.

## 1. Introduction

Self-sustained motion is defined as the periodic motion of an object with a fixed frequency and amplitude, which is the product of the system in response to a steady external stimulus [1,2,3,4,5]. Several appealing features are involved, for instance, the ability to harvest energy directly from the environment [6,7] as a driving force for the working equipment and the ability to achieve periodic motion without additional control devices [8,9]. They can reduce the system complexity to achieve intelligence and automation, and can reduce manpower consumption to save resources and improve efficiency. In addition, self-excited oscillations usually have good robustness [10]. These unique properties exhibited by self-oscillating systems have made them extensively applied in autonomous robots [11,12], motion [13,14,15], and automated transportation equipment [16,17,18].

A vast amount of recent work on self-oscillating systems functionalized with various active materials, have deepen the understanding of self-sustained mechanism and broaden the application scope. Active materials are a class of materials that respond to external stimuli by deformation and motion, which have been proven particularly useful in soft robotics and energy harvesting, including hydrogels [19,20], ionic gels [21,22], liquid crystal elastomers (LCEs) [23,24,25,26], etc. Numerous active materials have provided researchers the possibility to construct a variety of self-excited motion modes, e.g., bending [27,28,29], torsion [30,31], jumping [32,33,34], oscillation [35], and vibration [36,37]. These self-sustained motions usually arise from nonlinear feedback mechanisms that compensates for system damping dissipation through energy input [38,39,40], for instance, the self-shading mechanisms [41,42], the coupled chemical reaction and large deformation mechanisms [22], and the coupled air expansion and liquid column motion mechanisms [43,44].

As a type of stimuli, light offers several unique advantages in terms of its environmental friendliness and transient nature [45]. Photo-responsive materials, such as carbon nanotubes, graphene, and LCEs, possess sound light-driven deformation effects [46,47]. LCE is a network structure formed by cross-linking of liquid crystal molecules, which can undergo macroscopic deformation in response to various external stimuli such as electric field, temperature variation, magnetic field, and optical field [48]. In particular, a LCE containing azobenzenes, can deform under ultraviolet light irradiation. The variation in molecular configuration of azobenzenes from straight *trans* configuration to bent *cis* configuration can change the order degree of liquid crystal molecules, thus inducing strain in LCEs. Large reversible deformation and quick deformation response make LCEs appealing, and thereby open up the possibility of implementing various photo-responsive self-sustained oscillations, such as stretching [49], rolling [42], etc. These light-driven self-sustained systems can directly convert light energy into mechanical motion and have potential applications in energy harvesting [50,51], soft robotics [52], and micromachines [53].

Although a variety of LCE-based self-oscillation modes have been developed [54,55], the demand for constructing more novel self-oscillation systems still exists, which is beneficial to realize more functions. In this paper, a self-oscillating curling motion of LCE beam-mass system under steady light is creatively proposed. A LCE beam carrying two masses at two ends is placed on a table under irradiation of light sources at fixed height. Unlike other self-sustaining systems [56], the range of contact surface between the LCE beam and the tabletop is constantly changing during the self-sustaining curling under steady light, which is a complex dynamic boundary problem. In addition, the LCE beam placed on the tabletop is not fixedly connected to the tabletop and can move freely, which is expected to realize movements such as jumping.

The rest of current paper is organized as follows: In Section 2, based on the dynamic LCE model, we formulate and solve the governing equations of dynamics of the self-sustained curling LCE beam considering the dynamic boundary conditions. In Section 3, following the numerical calculations, two motion patterns of the LCE beam under steady light, i.e., stationary pattern and self-curling pattern, are introduced, and the mechanism of self-curling is subsequently explained. In Section 4, the effects of several key system parameters on the critical conditions, amplitude, and period of the self-curling of the LCE beam are investigated in detail. Finally, a summary is provided.

## 2. Model and Formulation

In this section, we first construct the self-curling LCE beam-mass system under steady light and then establish the theoretical model of the self-curling of the LCE beam considering dynamic boundary conditions based on the dynamic LCE plate model. The content mainly includes the dynamic equations of self-curling of LCE beam, the nondimensionalization of system parameters and the solution method of differential equations.

### 2.1. Dynamics of a LCE Beam-Mass System

**Figure 1:**
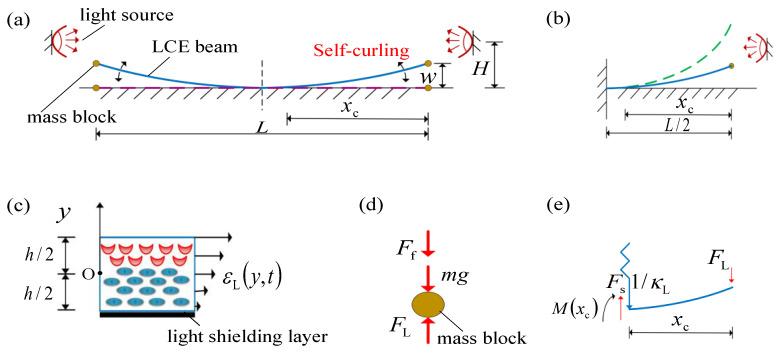
(**a**) Schematic diagram of the dynamic model of the LCE beam-mass system for self-sustained curling under steady light. (**b**) Equivalent schematic of the right half of the system in Figure 1a, where the LCE beam is partially in contact with the table. (**c**) Magnified cross-sectional view of the LCE beam, showing the optically-driven strain distribution on the beam section, with the lower surface of the LCE beam covered with a light-shielding layer. (**d**) Force analysis of the mass block at the end of the LCE beam, which is subjected to the mass gravity mg, the air damping force $F_{f}$, and the elastic force $F_{L}$ provided by the LCE beam. (**e**) Force analysis of the untouched part of the LCE beam, which is subjected to the elastic force $F_{L}$, the crosssection shear force $F_{s}$, and crosssection bending moment $M\left(x_{c}\right)$ provided by the touched part of LCE beam. Under steady light, the LCE beam can self-curl periodically.

Figure 1 plots the schematic diagram of a photo-responsive LCE beam-mass system that can sustain a self-curling motion under steady light. Initially, the LCE beam is placed on a horizontal table with mass blocks of mass *m* being attached to each end, as shown in Figure 1a. The LCE beam has length *L* and thickness *h*. The mass of the LCE beam is assumed to be much smaller than that of the mass block, and thus the inertia of the LCE beam is neglected. When a light source at fixed height *H* is introduced at each end of the LCE beam, only the upper surface of the LCE beam is exposed to light, and the LCE beam will bend upward as a result of the light-driven non-uniform contraction along the thickness direction, thereby in turn promoting the mass block to move upward. After the mass block passes the light source, the upper surface of the LCE beam is no longer illuminated, instead the lower surface is illuminated. Here, we assume that the lower surface is coated with a light-shielding layer. Subsequently, the LCE beam will bend back due to the recovery of light-driven contraction on the upper surface, leading to promote the mass block to move downward quickly. When the mass block moves rapidly downward below the light source, the upper surface of the LCE beam is exposed to light again, causing the mass block to be driven upward again subsequently. Ultimately, the LCE beam-mass system can undergo self-sustained curling motion under steady light. It is worth noting that the size of the contact surface between the LCE beam and the horizontal table is constantly varing during the curling process of the LCE beam, resulting in a dynamic boundary problem.

To describe the present state of the self-sustained curling LCE beam, we introduce the *y*-axis in the vertical direction. The position of the mass block at time *t* is denoted as $w\left(t\right)$. Given the symmetry of this problem, we select half of the LCE beam-mass system for analysis, as shown in Figure 1b. We emphasize that the intermediate symmetry plane of the LCE beam does not rotate, which is equivalent to a fixed end constraint (Figure 1b). The LCE beam bends in the light and acts elastic force $F_{L}$ on the mass block at the end, which is also subjected to gravity mg and damping force $F_{f}$, as shown in Figure 1d. Given that the eigenperiod of the LCE beam can be easily adjusted by changing the mass of the mass block and the bending stiffness of the LCE beam, for comparable eigenperiod and light response characteristic time, the dynamic equation governing the motion of the mass block are derived from mechanical equilibrium as follows [57],

(1) $$m\ddot{w}\left(t\right)=-mg+F_{f}\left(t\right)+F_{L}\left(t\right)$$

where g is the gravitational acceleration and $\ddot{w}\left(t\right)$ represents the acceleration of the mass block.

For simplicity, the damping force is assumed to be proportional to the velocity, namely

(2) $$F_{f}\left(t\right)=-\beta \dot{w}\left(t\right)$$

where β is the damping factor and $\dot{w}\left(t\right)$ represents the velocity of the mass block.

To calculate the elastic force of the LCE beam acting on the mass block, we need to first find the light-driven curvature $\kappa_{L}\left(t\right)$ of the LCE beam. Considering that the top or bottom surface of the beam is always illuminated or backlighted at the same time, the light illumination on either top or bottom surface of the LCE beam is assumed to be inhomogeneous for simplifying the modelling. For the illuminated LCE beam, the non-uniform light-driven strain varing along the thickness direction, is denoted as $\varepsilon_{L}\left(y,t\right)$. According to [57], the non-uniform light-driven strain will lead to the light-driven curvature of the LCE beam, namely

(3) $$\kappa_{L}\left(t\right)=\frac{\int_{-h/2}^{h/2}\varepsilon_{L}\left(y,t\right)ydy}{I_{Z}}$$

where $I_{Z}$ is the principal moment of inertia of the LCE beam section.

In general, at arbitrary instant of motion, the light-driven curvature of the LCE beam is $\kappa_{L}\left(t\right)$, the position of the mass block is $w\left(t\right)$, and the LCE beam is partially in contact with the table. The length of LCE beam leaving the table is $x_{c}\left(t\right)$, which varies continuously with the self-oscillating curling motion of the LCE beam and is to be quantified. The curvature at the contact junction is zero, so the crosssection bending moment $M\left(x_{c}\right)$ can be derived as

(4) $$M\left(x_{c}\right)=EI_{Z}\left(\kappa_{L}-0\right)$$

where E is the elastic modulus of LCE.

Likewise, we consider the equilibrium of the non-contact part of LCE beam with the table (Figure 1e), that is

(5) $$M\left(x_{c}\right)=F_{L}x_{c}\left(t\right)$$

Combining Equations (4) and (5), the non-contact length $x_{c}\left(t\right)$ of the LCE beam with the table is expressed as

(6) $$x_{c}\left(t\right)=\frac{EI_{Z}\kappa_{L}\left(t\right)}{F_{L}\left(t\right)}$$

Given the geometric relationship and deflection equation in Figure 1e, we can write

(7) $$\left(\frac{1}{\kappa_{L}\left(t\right)}-\sqrt{\frac{1}{\kappa_{L}^{2}\left(t\right)}-x_{c}^{2}\left(t\right)}\right)-w\left(t\right)=\frac{F_{L}\left(t\right)x_{c}^{3}\left(t\right)}{3EI_{Z}}$$

Combining Equations (6) and (7), the analytical formula of the elastic force $F_{L}$ is as follows

(8) $$F_{L}=\frac{\sqrt{2}EI_{Z}\kappa_{L}^{2}\left(t\right)}{\sqrt{-3-6\kappa_{L}\left(t\right)w\left(t\right)+3\sqrt{12\kappa_{L}\left(t\right)w\left(t\right)-4\kappa_{L}^{2}\left(t\right)w^{2}\left(t\right)+1}}}$$

Substituting Equations (2) and (8) into Equation (1), the dynamic equation of the mass block is obtained

(9) $$m\ddot{w}\left(t\right)=-mg-\beta \dot{w}\left(t\right)+\frac{\sqrt{2}EI_{Z}\kappa_{L}^{2}\left(t\right)}{\sqrt{-3-6\kappa_{L}\left(t\right)w\left(t\right)+3\sqrt{12\kappa_{L}\left(t\right)w\left(t\right)-4\kappa_{L}^{2}\left(t\right)w^{2}\left(t\right)+1}}}$$

It is worth noting that Equation (9) is determined by Equation (3). The light-driven contraction strain in Equation (3) is related to the *cis* number fraction of LCE material. For simplicity, the assumption is made that the light-driven contraction strain is proportional to the *cis* number fraction $\phi \left(y,t\right)$, i.e.,

(10) $$\varepsilon_{L}\left(y,t\right)=-C_{0}\phi \left(y,t\right)$$

where $C_{0}$ is the contraction coefficient.

### 2.2. Dynamic LCE Model

In the following, $\phi \left(y,t\right)$ is given by the dynamic LCE model. According to Corbett and Warner [58], the light intensity in LCE decreases exponentially along the depth direction. The LCE beam is uniformly illuminated and the light intensity at any position can be written as [58,59].

(11) $$I\left(y\right)=I_{0}\exp\left(-\frac{\left|y-y_{0}\right|}{d_{0}}\right)$$

where $I_{0}$ is the light intensity, $y_{0}$ is the vertical coordinate of exposed surface, and $d_{0}$ denotes the characteristic penetration depth.

The *cis*-number fraction is generally relevant to the thermal excitation from *trans* to *cis*, the thermally driven relaxation from *cis* to *trans*, as well as the light-driven isomerization. The thermal excitation from *trans* to *cis* is often negligible and $\phi \left(y,t\right)$ can be described by [60,61,62]

(12) $$\frac{\partial \phi }{\partial t}=\eta_{0}I\left(1-\phi \right)-T_{0}^{-1}\phi$$

where $T_{0}$ denotes the thermal relaxation time from the *cis* to *trans* state and $\eta_{0}$ represents the light-absorption constant. By considering an initial condition and assuming $T_{0}\eta_{0}I<<1$ [61], the solution to Equation (12) can be approximately expressed as

(13) $$\phi \left(y,t\right)=\eta_{0}T_{0}I+\left(\phi_{0}-\eta_{0}T_{0}I\right)\exp\left(-\frac{t}{T_{0}}\right)$$

in which, $\phi_{0}$ is the initial *cis* number fraction.

In this paper, the LCE beam switches between light irradiation and darkness. For Case I that the LCE beam is in the illumination zone with initial $\phi_{0}=0$, the *cis* number fractions can be reduced to

(14) $$\phi_{0}\left(t\right)=\eta_{0}T_{0}I_{0}\left[1-\exp\left(-\frac{t_{1}}{\tau_{0}}\right)\right]$$

For Case II that the LCE beam is in the illumination zone switched from the dark zone with transient $\phi_{0}=\phi_{\mathrm{dark}}$, the *cis* number fractions can be reduced to

(15) $$\phi_{0}\left(t\right)=\eta_{0}T_{0}I_{0}+\left(\phi_{\mathrm{dark}}-\eta_{0}T_{0}I_{0}\right)\exp\left(-\frac{t_{2}}{\tau_{0}}\right)$$

For Case III that the LCE beam is in the dark zone ($I_{0}=0$), switched from the illumination zone with transient $\phi_{0}=\phi_{\mathrm{illum}}$, the *cis* number fractions can be reduced to

(16) $$\phi_{0}\left(t\right)=\phi_{\mathrm{illum}}\exp\left(-\frac{t_{3}}{\tau_{0}}\right)$$

In Equations (14)–(16), ${\bar{t}}_{1}$, ${\bar{t}}_{2}$ and ${\bar{t}}_{3}$ are the durations of current process, respectively, $\phi_{\mathrm{dark}}$ and $\phi_{\mathrm{illum}}$ are the transient number fractions of *cis*-isomers at the instant of switching from the dark zone into the illumination zone, and from the illumination zone into the dark zone, respectively.

We remark that $\phi \left(y,t\right)$ is always linearly proportional to $I\left(y\right)$ for $\phi_{0}=0$. Therefore, we can rewrite Equation (13) as

(17) $$\phi \left(y,t\right)=\phi_{0}\left(t\right)\exp\left(-\frac{h/2-y}{d_{0}}\right),\mathrm{for}-\frac{h}{2}\leq y\leq \frac{h}{2}$$

in which, $\phi_{0}\left(t\right)$ is the *cis* number fraction on the illuminated surface.

Combining Equations (3), (10) and (17), we can obtain the curvature $\kappa_{L}\left(t\right)$ formed at the end mass of the LCE beam during self-oscillation, being proportional to the *cis* number fraction $\phi_{0}\left(t\right)$, as

(18) $$\kappa_{L}\left(t\right)=A\phi_{0}\left(t\right)$$

where A is the curvature coefficient, namely

(19) $$A=\frac{-C_{0}d_{0}\left(h/2-d_{0}\right)\left(1+e^{-h∕d_{0}}\right)}{I_{Z}}$$

### 2.3. Nondimensionalization and Solution

For convenience, the dimensionless quantities are introduced as follows: ${\overset{¯}{I}}_{0}=\eta_{0}I_{0}T_{0}$, $\overset{¯}{t}=\frac{t}{T_{0}}$, $\overset{¯}{\beta }=\frac{\beta T_{0}}{m}$, $\overset{¯}{H}=\frac{H}{L}$, $\overset{¯}{g}=\frac{gT_{0}^{2}}{L}$, $\overset{¯}{w}=\frac{w}{L}$, $\overset{¯}{E}=\frac{EI_{Z}T_{0}^{2}}{mL^{3}}$, ${\overset{¯}{\kappa }}_{L}=\kappa_{L}L$, and $\overset{¯}{A}=\frac{-C_{0}d_{0}L^{2}\left(h/2-d_{0}\right)\left(1+e^{-h∕d_{0}}\right)}{I_{Z}}$. From Equation (8), the dimensionless elastic force can be expressed as

(20) $${\overset{¯}{F}}_{L}=\frac{\sqrt{2}\overset{¯}{E}{\overset{¯}{\kappa }}_{L}^{2}}{\sqrt{-3-6\left({\overset{¯}{\kappa }}_{L}\overset{¯}{w}\right)+3\sqrt{12\left({\overset{¯}{\kappa }}_{L}\overset{¯}{w}\right)-4{\left({\overset{¯}{\kappa }}_{L}\overset{¯}{w}\right)}^{2}+1}}}$$

The governing Equation (9) can be nondimensionalized with the following

(21) $$-\overset{¯}{g}+\frac{\sqrt{2}\overset{¯}{E}{\overset{¯}{\kappa }}_{L}^{2}}{\sqrt{-3-6\left({\overset{¯}{\kappa }}_{L}\overset{¯}{w}\right)+3\sqrt{12\left({\overset{¯}{\kappa }}_{L}\overset{¯}{w}\right)-4{\left({\overset{¯}{\kappa }}_{L}\overset{¯}{w}\right)}^{2}+1}}}-\overset{¯}{\beta }\overset{¯}{\dot{w}}=\overset{¯}{\ddot{w}}$$

From Equations (15) and (17)–(19), the light-driven curvature can be rewritten as follows, for Case I,

(22) $$\kappa_{L}\left(\overset{¯}{t}\right)=\overset{¯}{A}{\overset{¯}{I}}_{0}\left[1-\exp\left(-{\overset{¯}{t}}_{1}\right)\right]$$

for Case II,

(23) $$\kappa_{L}\left(\overset{¯}{t}\right)=\overset{¯}{A}{\overset{¯}{I}}_{0}-\left(\kappa_{\mathrm{dark}}-\overset{¯}{A}{\overset{¯}{I}}_{0}\right)\exp\left(-{\overset{¯}{t}}_{2}\right)$$

and for Case III,

(24) $$\kappa_{L}\left(\overset{¯}{t}\right)=\kappa_{\mathrm{illum}}\exp\left(-{\overset{¯}{t}}_{3}\right)$$

where $\kappa_{\mathrm{dark}}$ and $\kappa_{\mathrm{illum}}$ are the light-driven curvature at the instant of switching from the dark zone into the illumination zone, and from the illumination zone into the dark zone, respectively. Since ${\overset{¯}{t}}_{1}$, ${\overset{¯}{t}}_{2}$ and ${\overset{¯}{t}}_{3}$ are the durations of current process, light-driven curvature $\kappa_{L}$ is process-related and time-dependent.

The initial condition of the LCE beam is as follows: when $\overset{¯}{t}=0$,

(25) $$\overset{¯}{w}={\overset{¯}{w}}_{0},\overset{¯}{\dot{w}}={\overset{¯}{\dot{w}}}_{0}$$

Taking into account the dimensionless parameters $\overset{¯}{H}$, ${\overset{¯}{I}}_{0}$, $\overset{¯}{A}$, $\overset{¯}{E}$, $\overset{¯}{\beta }$, $\overset{¯}{g}$, and $\overset{¯}{\dot{w}}$, Equations (20)–(25) govern the motion of the LCE beam-mass system under steady light. To solve the complex differential Equation (21) with variable coefficients, we perform numerical calculations in the software Matlab based on the fourth-order Runge-Kutta method. In the calculation, we give the LCE beam an initial displacement. For the previous position ${\overset{¯}{w}}_{i-1}$ and the previous curvature $\kappa_{L}{}_{\left(i-1\right)}$, we can calculate the corresponding elastic force ${\overset{¯}{F}}_{L\left(i-1\right)}$according to Equation (20). The current position ${\overset{¯}{w}}_{i}$ can be further calculated from Equation (21), and the current curvature $\kappa_{L}{}_{i}$ can be calculated from Equations (22)–(24). When the current position ${\overset{¯}{w}}_{i}<\overset{¯}{H}$, the upper surface of the LCE beam is in the light irradiation; when the current position ${\overset{¯}{w}}_{i}>\overset{¯}{H}$, the upper surface of the LCE beam is in the darkness. Based on the current curvature $\kappa_{L}{}_{i}$, we can obtain the current elastic force ${\overset{¯}{F}}_{L\left(i\right)}$ by Equation (20). Then we continue to obtain the position ${\overset{¯}{w}}_{\left(i+1\right)}$ and curvature ${\overset{¯}{\kappa }}_{L\left(i+1\right)}$ of the LCE beam in turn from Equations (22)–(25). Through iterative calculations, the time history of the position of the self-curling LCE beam can be obtained and the effects of different parameters on its self-curling can be further investigated.

## 3. Two Motion Patterns and Mechanism of the Self-Curling

Considering the above governing equations, we investigate the dynamic behavior of the self-oscillating curling of the LCE beam under steady light through numerical calculations. We first introduce two motion patterns, namely stationary pattern and self-curling pattern; then the corresponding mechanism of self-curling is elaborated by parametric analysis.

### 3.1. Two Motion Patterns

To study the self-oscillating curling motion of the LCE beam, we first need to determine the dimensionless parameters in the theoretical model. Taking data from the existing experiments [12,63,64], the material properties and geometric parameters of the system, and the corresponding dimensionless parameters are respectively listed in Table 1 and Table 2. The following parameter values are used in current paper to study the self-curling of the LCE beam under steady light.

**Figure 2:**
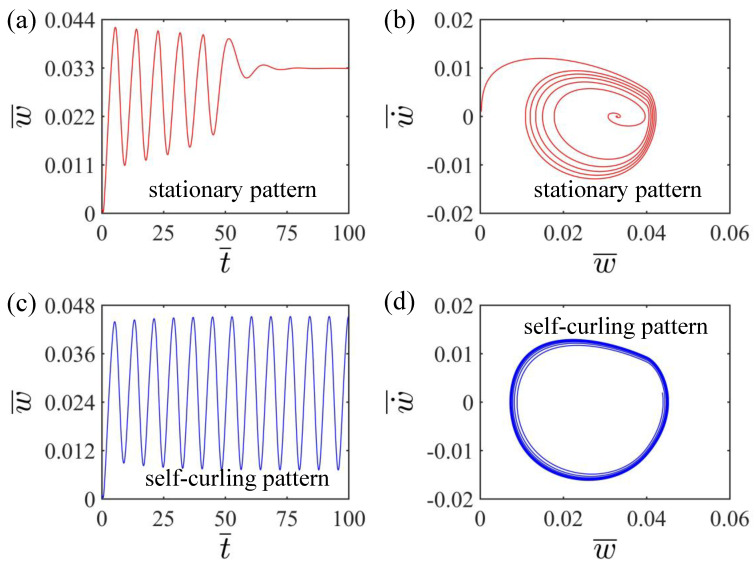
(**a**) Time history and (**b**) phase trajectory diagram of the stationary pattern of the LCE beam-mass system for $\overset{¯}{H}=0.04$, $\overset{¯}{A}=0.36$, ${\overset{¯}{I}}_{0}=0.058$, $\overset{¯}{E}=2.4$, $\overset{¯}{g}=0.015$, $\overset{¯}{\beta }=0.30$ and $\overset{¯}{\dot{w}}=0$. (**c**) Time history and (**d**) phase trajectory diagram of the self-curling pattern of the LCE beam-mass system for $\overset{¯}{H}=0.04$, $\overset{¯}{A}=0.36$, ${\overset{¯}{I}}_{0}=0.06$, $\overset{¯}{E}=2.4$, $\overset{¯}{g}=0.015$, $\overset{¯}{\beta }=0.30$ and $\overset{¯}{\dot{w}}=0$. Two motion patterns exist for the LCE beam-mass system under steady light: the stationary pattern and the self-curling pattern.

Two typical motion patterns as well as the corresponding phase trajectories of the LCE beam under steady light are given in Figure 2. The numerical calculation results show the existence of two motion patterns of the LCE beam: the stationary pattern and the self-curling pattern. When $\overset{¯}{A}=0.36$, $\overset{¯}{E}=2.4$, $\overset{¯}{g}=0.015$, $\overset{¯}{\beta }=0.30$, $\overset{¯}{\dot{w}}=0$, $\overset{¯}{H}=0.04$, and ${\overset{¯}{I}}_{0}=0.058$, the LCE beam under steady light initially bends upward due to the light-driven contraction in the upper surface of LCE beam exposed to light. And after the mass block passes cross the light source, the LCE beam is promoted to bend downward due to the recovery of the light-driven contraction in the upper surface of LCE beam in darkness. Due to the air damping, the oscillation amplitude of the LCE beam decreases continuously and finally remains stable, which is called the stationary pattern, as shown in Figure 2a,b. When $\overset{¯}{A}=0.36$, $\overset{¯}{E}=2.4$, $\overset{¯}{g}=0.015$, $\overset{¯}{\beta }=0.30$, $\overset{¯}{\dot{w}}=0$, $\overset{¯}{H}=0.04$, and ${\overset{¯}{I}}_{0}=0.06$, the oscillation amplitude of the LCE beam exhibits a slight increasing trend and the beam eventually presents a continuous oscillation at a constant amplitude, which is named as the self-curling pattern, as shown in Figure 2c,d. Similar to other self-oscillating systems, the LCE beam can undergo a self-oscillation pattern with a steady light source, which is generally because of the energy compensation between the light energy and the energy dissipated by air damping, so as to maintain the self-oscillation. The following part will focus on the mechanism of the self-oscillating curling in detail.

### 3.2. Mechanism of Self-Curling

**Figure 3:**
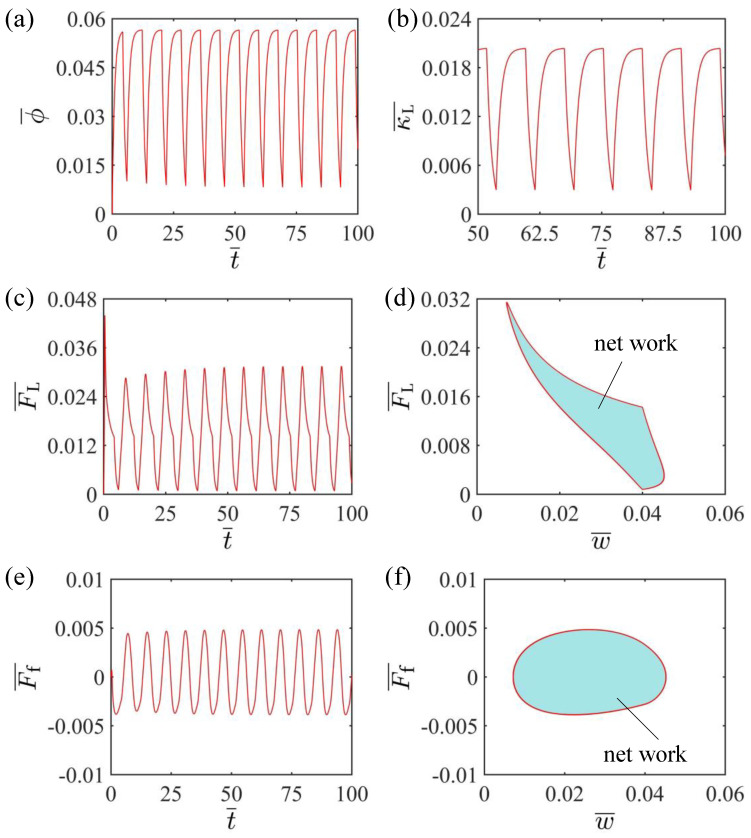
(**a**) Time dependence of the *cis*-number fraction of the LCE beam; (**b**) Time dependence of the curvature of the LCE beam; (**c**) Time dependence of the elastic force of the LCE beam; (**d**) Dependence of the elastic force on the displacement of the mass block. (**e**) Time dependence of the damping force; (**f**) Dependence of the damping force on the displacement of the mass block. The area enclosed in Figure 3d indicates the net work done by the elastic force, which is equal to the energy dissipated by the damping, i.e., the self-curling pattern is maintained.

To investigate the self-oscillating curling mechanism of the LCE beam, Figure 3 displays the evolutions of several key parameters for the self-curling pattern of the LCE beam in Figure 2c,d. Figure 3a plots the time dependence of the *cis* number fraction of the LCE material, showing a periodic variation with time. Figure 3b plots the time dependence of the curvature of the LCE beam, which also varies with time periodically. Figure 3c plots the time dependence of the elastic force acting on the mass block from the LCE beam, and it is evident that the elastic force also varies with time in a periodic manner. As shown in Figure 3d, the dependence of the elastic force on the displacement of the mass block is plotted. There forms a closed curve in one cycle. The area enclosed by this closed curve represents the net work done by the elastic force, calculated as $2.68\times 10^{-4}$. Meanwhile, the time dependence of the damping force acting on the mass block is plotted in Figure 3e, and the results show that the damping force varies periodically with time. The dependence of the damping force on the displacement of the mass is plotted as shown in Figure 3f. There also forms a closed curve in one cycle. The area enclosed by this closed curve represents the damping dissipation energy, which is calculated as $2.68\times 10^{-4}$. It is equal to the net work done by the elastic force so that a continuous and stable self-oscillating curling state can be maintained.

## 4. Parametric Study

The following dimensionless parameters exist in the above theoretical model: $\overset{¯}{H}$, $\overset{¯}{A}$, $\overset{¯}{E}$, ${\overset{¯}{I}}_{0}$, $\overset{¯}{g}$ and $\overset{¯}{\beta }$. In current section, we will investigate the effects of these key system parameters on the critical conditions, the amplitude and period of the self-oscillating curling of the LCE beam-mass system, expecting to guide its applications in soft robotics, energy harvesters, micro-instrumentation, and other fields.

### 4.1. Effect of Dimensionless Light Source Height

**Figure 4:**
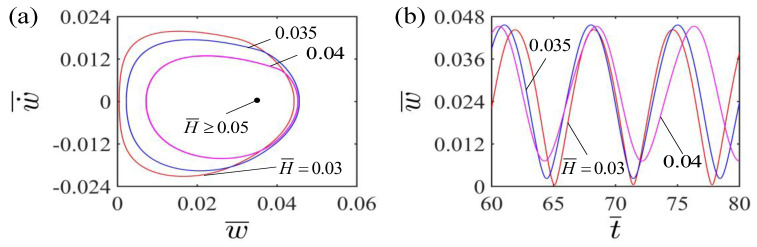
Effect of dimensionless light source height on the self-curling of the LCE beam-mass system for the other parameters $\overset{¯}{A}=0.36$, $\overset{¯}{E}=2.4$, ${\overset{¯}{I}}_{0}=0.06$, $\overset{¯}{g}=0.015$, $\overset{¯}{\beta }=0.30$ and $\overset{¯}{\dot{w}}=0$. (**a**) Limit cycles; (**b**) Time histories for different light source heights $\overset{¯}{H}$. Both amplitude and period of the self-curling increase as the light source height $\overset{¯}{H}$ increases.

Figure 4 illustrates the effect of dimensionless light source height on the self-curling of the LCE beam. In the calculation, we set other parameters as $\overset{¯}{A}=0.36$, $\overset{¯}{E}=2.4$, ${\overset{¯}{I}}_{0}=0.06$, $\overset{¯}{g}=0.015$, $\overset{¯}{\beta }=0.30$ and $\overset{¯}{\dot{w}}=0$. Figure 4a plots the limit cycles for self-curling of the LCE beam at different dimensionless light source heights. The critical light source height $\overset{¯}{H}$ for the existence of self-curling is 0.05. When the dimensionless light source height exceeds the critical value, the LCE beam finally stays in the static equilibrium position and maintains the steady state, which is the stationary pattern. For $\overset{¯}{H}=0.03$, $\overset{¯}{H}=0.035$ and $\overset{¯}{H}=0.04$, the LCE beam is capable of self-oscillating curling. Figure 4b presents the effect of different dimensionless light source heights on the amplitude and period of the self-curling. Both amplitude and period increase with the increase of the dimensionless light source height. This is attributed to the fact that the higher the height $\overset{¯}{H}$ of the light source, the longer duration of the LCE beam being irradiated during the self-curling, the more the light energy input, and the greater the amplitude and period of the self-curling of LCE beam. In practical application, the light source can be placed further away from the LCE beam to meet the requirements of working conditions, which will complicate the problem more. However, we believe that the position of the light source has no qualitative impact on the results, only quantitative impact.

### 4.2. Effect of Dimensionless Curvature Coefficient

**Figure 5:**
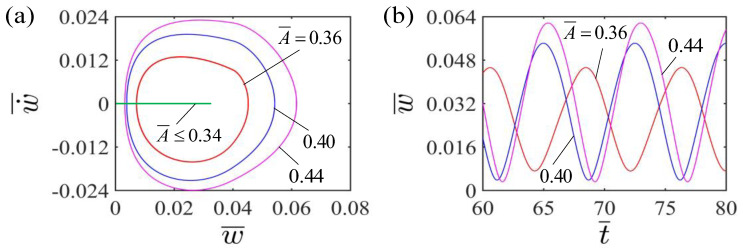
Effect of dimensionless curvature coefficient on the self-curling of the LCE beam-mass system for the other parameters $\overset{¯}{H}=0.04$, $\overset{¯}{E}=2.4$, ${\overset{¯}{I}}_{0}=0.06$, $\overset{¯}{g}=0.015$, $\overset{¯}{\beta }=0.30$ and $\overset{¯}{\dot{w}}=0$. (**a**) Limit cycles; (**b**) Time histories for different curvature coefficients $\overset{¯}{A}$. As the dimensionless curvature coefficient increases, the self-curling amplitude increases significantly, while the self-curling period remains almost constant.

Figure 5 shows the effect of different curvature coefficients $\overset{¯}{A}$ on the self-curling of the LCE beam. In the calculation, we set the other parameters $\overset{¯}{H}=0.04$, $\overset{¯}{E}=2.4$, ${\overset{¯}{I}}_{0}=0.06$, $\overset{¯}{g}=0.015$, $\overset{¯}{\beta }=0.30$ and $\overset{¯}{\dot{w}}=0$. Figure 5a depicts the limit cycles of self-curling for LCE beams with different dimensionless curvature coefficients. The critical curvature coefficient $\overset{¯}{A}$ for the LCE beam to undergo self-curling pattern is about 0.34. When the dimensionless curvature coefficient is below the critical value, the light energy input is less than the energy consumed by the damping, so that the LCE beam finally stays in the static equilibrium position. For $\overset{¯}{A}=0.36$, $\overset{¯}{A}=0.4$ and $\overset{¯}{A}=0.44$, LCE beam can occur with self-oscillating curling. Further, Figure 5b displays the effect of different curvature coefficients $\overset{¯}{A}$ on the self-curling amplitude and period of the LCE beam. As the dimensionless curvature coefficient increases, the self-curling amplitude increases significantly. This is attributable to the fact that the larger the curvature coefficient is, the more the work done by the LCE beam to the mass block, and in turn the greater the amplitude. Meanwhile, the self-curling period remains almost constant as the dimensionless curvature coefficient increases. This is because that the dimensionless curvature coefficient only reflects the deformation without changing the natural frequency of the system.

### 4.3. Effect of Dimensionless Light Intensity

**Figure 6:**
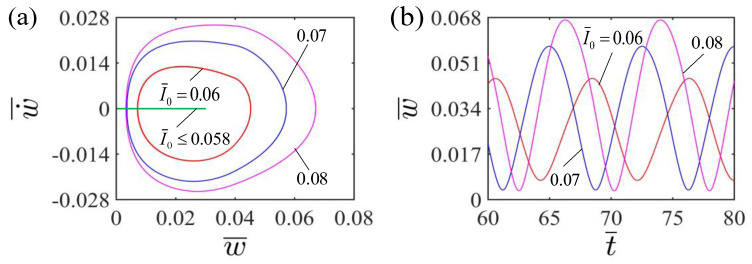
Effect of dimensionless light intensity on the self-curling of the LCE beam-mass system for the other parameters $\overset{¯}{H}=0.04$, $\overset{¯}{A}=0.36$, $\overset{¯}{E}=2.4$, $\overset{¯}{g}=0.015$, $\overset{¯}{\beta }=0.30$ and $\overset{¯}{\dot{w}}=0$. (**a**) Limit cycles; (**b**) Time histories for different light intensities ${\overset{¯}{I}}_{0}$. As the light intensity ${\overset{¯}{I}}_{0}$ increases, the self-curling amplitude displays a significant increase, while the self-curling period remains almost unchanged. Figure 6 presents the effect of different light intensities ${\overset{¯}{I}}_{0}$ on the self-curling of the LCE beam. In the calculation, we set the other parameters $\overset{¯}{H}=0.04$, $\overset{¯}{A}=0.36$, $\overset{¯}{E}=2.4$, $\bar{g}=0.015$, $\overset{¯}{\beta }=0.30$ and $\overset{¯}{\dot{w}}=0$.

Figure 6a plots the limit cycles of self-curling for LCE beam at different light intensities ${\overset{¯}{I}}_{0}$. The critical light intensity ${\overset{¯}{I}}_{0}$ for the occurrence of self-curling pattern of the LCE beam is 0.058. When the dimensionless light intensity is less than the critical value, the LCE beam finally stays in the static equilibrium position and maintains a steady state. This is because the mechanical energy of the system converted from the light energy is not sufficient enough to compensate for the energy dissipation caused by damping. For ${\overset{¯}{I}}_{0}=0.06$, ${\overset{¯}{I}}_{0}=0.07$ and ${\overset{¯}{I}}_{0}=0.08$, the self-curling pattern of the LCE beam will emerge. Figure 6b illustrates the effect of dimensionless light intensity on the self-curling amplitude and period of the LCE beam. The amplitude displays a significant increase with the increasing light intensity ${\overset{¯}{I}}_{0}$. This is because the greater the light intensity ${\overset{¯}{I}}_{0}$, the more the light energy is converted into the mechanical energy of the system, consequently the greater the amplitude. Similarly, the period remains almost unchanged with the increase of dimensionless light intensity. This is because that the dimensionless light intensity does not affect the natural frequency of the system.

### 4.4. Effect of Dimensionless Elasticity Modulus

**Figure 7:**
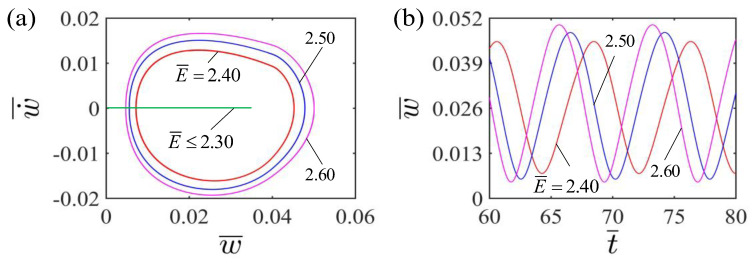
Effect of dimensionless elastic modulus on the self-curling of the LCE beam-mass system for the other parameters $\overset{¯}{H}=0.04$, $\overset{¯}{A}=0.36$, ${\overset{¯}{I}}_{0}=0.06$, $\overset{¯}{g}=0.015$, $\overset{¯}{\beta }=0.30$ and $\overset{¯}{\dot{w}}=0$. (**a**) Limit cycles; (**b**) Time histories for different elastic moduli $\overset{¯}{E}$. As the elastic modulus $\overset{¯}{E}$ increases, the self-curling amplitude increases significantly, while the self-curling period is suppressed.

Figure 7 shows the effect of the dimensionless elastic modulus on the self-curling of the LCE beam. In the calculation, we set the other parameters to $\overset{¯}{H}=0.04$, $\overset{¯}{A}=0.36$, ${\overset{¯}{I}}_{0}=0.06$, $\overset{¯}{g}=0.015$, $\overset{¯}{\beta }=0.30$ and $\overset{¯}{\dot{w}}=0$. Figure 7a depicts the limit cycles of the self-curling of the LCE beam for different elastic moduli $\overset{¯}{E}$. There exists a critical elastic modulus $\overset{¯}{E}$ for the self-curling of the LCE beam, being about 2.3. When the dimensionless elastic modulus is below the critical value, the LCE beam finally stays in the static equilibrium position and maintains a stable stationary pattern. This is because the smaller the elastic modulus $\overset{¯}{E}$, the softer the LCE beam is, thus the mechanical energy of the system arising from the conversion of light energy is unable to compensate for the energy lost in damping to maintain the self-curling. For $\overset{¯}{E}=2.40$, $\overset{¯}{E}=2.50$ and $\overset{¯}{E}=2.60$, the self-curling pattern of the LCE beam will occur, with their limit cycles being plotted in Figure 7a. Meanwhile, Figure 7b shows the effect of dimensionless elastic modulus on the amplitude and period of the self-curling. As the dimensionless elastic modulus increases, the amplitude increases significantly while the period decreases. This is due to the fact that a larger dimensionless elastic modulus will provide a larger elastic force and a smaller eigenperiod, which is consistent with physical intuition.

### 4.5. Effect of Dimensionless Gravitational Acceleration

**Figure 8:**
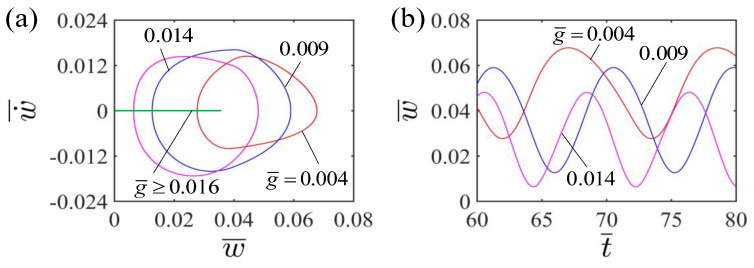
Effect of dimensionless gravitational acceleration on the self-curling of the LCE beam-mass system for the other parameters $\overset{¯}{H}=0.04$, $\overset{¯}{A}=0.36$, $\overset{¯}{E}=2.4$, ${\overset{¯}{I}}_{0}=0.06$, $\overset{¯}{\beta }=0.30$ and $\overset{¯}{\dot{w}}=0$. (**a**) Limit cycles; (**b**) Time histories for different gravitational accelerations $\overset{¯}{g}$. As the gravitational acceleration $\overset{¯}{g}$ increases, the self-curling amplitude exhibits a considerable decrease, and the self-curling period is suppressed. Figure 8 presents the effect of dimensionless gravitational acceleration on the self-curling of the LCE beam. In the calculation, we set the other parameters $\overset{¯}{H}=0.04$, $\overset{¯}{A}=0.36$, $\overset{¯}{E}=2.4$, ${\overset{¯}{I}}_{0}=0.06$, $\overset{¯}{\beta }=0.30$ and $\overset{¯}{\dot{w}}=0$.

Figure 8a displays the limit cycles of the self-curling of the LCE beam for different gravitational accelerations $\overset{¯}{g}$. There exists a critical gravitational acceleration $\overset{¯}{g}$ value of about 0.016 to trigger the LCE beam to exhibit self-oscillating curling. When the dimensionless gravitational acceleration exceeds the critical value, the LCE beam will maintain the static stationary pattern because the energy input is not enough to compensate for the energy loss when the dimensionless gravitational acceleration is large. For $\overset{¯}{g}=0.004$, $\overset{¯}{g}=0.009$ and $\overset{¯}{g}=0.014$, the self-curling can emerge in the LCE beam. Meanwhile, Figure 8b shows the effect of gravitational acceleration $\overset{¯}{g}$ on the self-curling amplitude and period of the LCE beam. The self-curling amplitude exhibits a considerable decrease with the increasing dimensionless gravitational acceleration, which is attributed to the fact that a larger gravitational acceleration $\overset{¯}{g}$ will lead to less work done by the LCE beam on the mass block. Meanwhile, the self-curling period is also suppressed by the dimensionless gravitational acceleration, which is consistent with physical intuition. Considering $\overset{¯}{g}=gT_{0}^{2}/L$, the dimensionless $\overset{¯}{g}$ can be easily tuned by changing the length of LCE beam to satisfy the requirement of applications.

### 4.6. Effect of Dimensionless Damping Factor

**Figure 9:**
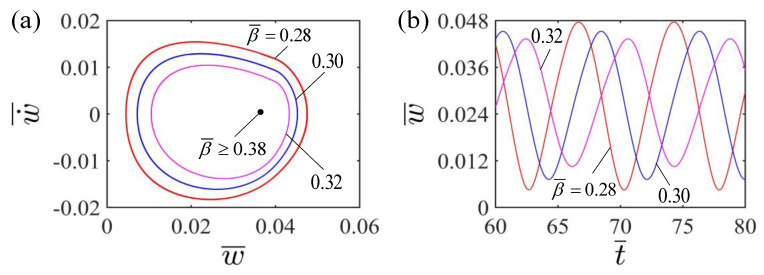
Effect of dimensionless damping factor on the self-curling of the LCE beam-mass system for the other parameters $\overset{¯}{H}=0.04$, $\overset{¯}{A}=0.36$, $\overset{¯}{E}=2.4$, ${\overset{¯}{I}}_{0}=0.06$, $\overset{¯}{g}=0.015$ and $\overset{¯}{\dot{w}}=0$. (**a**) Limit cycles; (**b**) Time histories for different damping factors $\overset{¯}{\beta }$. As the damping factor $\overset{¯}{\beta }$ increases, the self-curling amplitude decreases significantly, while the self-curling period remains almost constant.

Figure 9 shows the effect of the damping factor $\overset{¯}{\beta }$ on the self-curling of the LCE beam. In the calculation, we set the other parameters $\overset{¯}{H}=0.04$, $\overset{¯}{A}=0.36$, $\overset{¯}{E}=2.4$, ${\overset{¯}{I}}_{0}=0.06$, $\overset{¯}{g}=0.015$ and $\overset{¯}{\dot{w}}=0$. Figure 9a plots the limit cycles of self-curling for LCE beams with different damping factors $\overset{¯}{\beta }$. A critical damping factor $\overset{¯}{\beta }$ valued about 0.38 exists for the occurrence of the self-curling for LCE beams. When the dimensionless damping factor is greater than the critical value, the damping energy dissipation exceeds the mechanical energy of the system converted from light energy, so that the LCE beam will eventually maintain stationary. For $\overset{¯}{\beta }=0.28$, $\overset{¯}{\beta }=0.30$ and $\overset{¯}{\beta }=0.32$, the LCE beam undergoes self-curling pattern. Meanwhile, Figure 9b displays the effect of the damping factor $\overset{¯}{\beta }$ on the self-curling amplitude and period of the LCE beam. As the dimensionless damping factor increases, the amplitude decreases significantly. This is due to the fact that the larger the dimensionless damping factor, the more energy the damping consumes, and as a result, the smaller the amplitude. Meanwhile, the period remains almost constant with increasing dimensionless damping factor, for the dimensionless damping factor hardly changes the natural frequency of the system. This result is similar to other self-oscillating systems [27,28,29,30,31,32,33,34,35].

## 5. Conclusions

Self-oscillation can actively draw energy from the environment to maintain its own motion and has important application prospects in energy harvesting, soft robotics, and micromachines. In current paper, we propose a LCE beam-mass system that can exhibit self-curling pattern under steady light, among which the LCE beam carrying two masses at two ends is placed on a tabletop under irradiation of light sources at fixed height. Unlike other self-sustained systems, the varing contact surface between the LCE beam and the tabletop indicates the dynamic boundary problem. Based on the existing dynamic LCE model, we developed a nonlinear dynamic model for the self-oscillating curling of the LCE beam-mass system under steady light. Numerically solved by the fourth-order Runge-Kutta method, the computational results demonstrate that two types of motion patterns exist, namely the stationary pattern and the self-curling pattern. The self-curling of the LCE beam originates from its periodic light-driven contraction and relaxation. Further, we investigate the effects of different dimensionless parameters on the critical conditions, the amplitude, and the period of self-curling of the LCE beam. The results show that the increases in light source height, curvature coefficient, light intensity, and elastic modulus, will promote the self-curling amplitude to be increased, while the gravitational acceleration and damping factor do the opposite. The light source height promotes the self-curling period while the elastic modulus suppresses the period, while the other parameters do not affect the self-curling period. In general, the above results are expected to deepen the understanding of the self-oscillation phenomenon and provide design ideas for applications such as autonomous robotics, sensors, energy harvesting, and microinstrumentation.

## Figures and Tables

**Table 1:** Material properties and geometric parameters.

Parameter	Definition	Value	Units
$C_{0}$	contraction coefficient	0~0.5	/
$T_{0}$	*trans*-to-*cis* thermal relaxation time	0.001~0.1	s
$I_{0}$	light intensity	0~10	kW/m^2^
$\eta_{0}$	light-absorption constant	0.0001	m^2^/(s·W)
*m*	mass of the mass block	0.001	kg
*E*	elastic modulus of LCE material	1~10	MPa
*β*	damping factor	0~1	kg/s
g	gravitational acceleration	10	m/s^2^
$d_{0}$	characteristic penetration depth	10^−5^	m
h	thickness of LCE beam	10^−4^	m
$I_{Z}$	principal moment of inertia	10^−7^	$m^{4}$
*L*	length of LCE beam	0.01~0.02	m
*H*	light source height	0~0.002	m

**Table 2:** Dimensionless parameters.

Parameter	$\overset{¯}{H}$	$\overset{¯}{A}$	${\overset{¯}{I}}_{0}$	$\overset{¯}{E}$	$\overset{¯}{\beta }$	$\overset{¯}{g}$
Value	0~0.1	0~0.5	0~0.1	0~10	0~0.5	10^−3^~10

## Data Availability

The data that support the findings of this study are available upon reasonable request from the authors.
